# Search for β_2_ Adrenergic Receptor Ligands by Virtual Screening via Grid Computing and Investigation of Binding Modes by Docking and Molecular Dynamics Simulations

**DOI:** 10.1371/journal.pone.0107837

**Published:** 2014-09-17

**Authors:** Qifeng Bai, Yonghua Shao, Dabo Pan, Yang Zhang, Huanxiang Liu, Xiaojun Yao

**Affiliations:** 1 Department of Chemistry, Lanzhou University, Lanzhou, China; 2 School of Information Science & Engineering, Lanzhou University, Lanzhou, China; 3 School of Pharmacy, Lanzhou University, Lanzhou, China; 4 School of Basic Medical Sciences, Lanzhou University, Lanzhou, China; 5 State Key Laboratory of Quality Research in Chinese Medicine, Macau Institute for Applied Research in Medicine and Health, Macau University of Science and Technology, Taipa, Macau, China; Medical School of Hannover, Germany

## Abstract

We designed a program called MolGridCal that can be used to screen small molecule database in grid computing on basis of JPPF grid environment. Based on MolGridCal program, we proposed an integrated strategy for virtual screening and binding mode investigation by combining molecular docking, molecular dynamics (MD) simulations and free energy calculations. To test the effectiveness of MolGridCal, we screened potential ligands for β_2_ adrenergic receptor (β_2_AR) from a database containing 50,000 small molecules. MolGridCal can not only send tasks to the grid server automatically, but also can distribute tasks using the screensaver function. As for the results of virtual screening, the known agonist BI-167107 of β_2_AR is ranked among the top 2% of the screened candidates, indicating MolGridCal program can give reasonable results. To further study the binding mode and refine the results of MolGridCal, more accurate docking and scoring methods are used to estimate the binding affinity for the top three molecules (agonist BI-167107, neutral antagonist alprenolol and inverse agonist ICI 118,551). The results indicate agonist BI-167107 has the best binding affinity. MD simulation and free energy calculation are employed to investigate the dynamic interaction mechanism between the ligands and β_2_AR. The results show that the agonist BI-167107 also has the lowest binding free energy. This study can provide a new way to perform virtual screening effectively through integrating molecular docking based on grid computing, MD simulations and free energy calculations. The source codes of MolGridCal are freely available at http://molgridcal.codeplex.com.

## Introduction

Grid computing can collect computer resources from different locations to deal with data more effectively and rapidly [1]. This advantage of grid computing has led to its wide and successful applications in many different fields, such as Hadron Collider [2], nuclear magnetic resonance (NMR) [3], image analysis [4] and so on. Especially, grid computing was successfully used in huge microbial sequence analysis [5]–[7] and biology medicine research [8]–[12]. Grid computing also played an important role in molecular simulations and computer aided drug discovery [13]. For example, the grid computing framework of folding@home [14] was used to simulate protein folding process by using the idle computer resources. Furthermore, the successful applications of grid computing in molecular docking suggested that virtual screening could also be integrated into grid computing environment [15] due to the fact that many molecular docking programs [16]–[21], and small molecule databases [22]–[24] are available. The screensaver project of grid computing could supply enough computing resource to perform effective virtual screening [25]. The use of virtual screening [26]–[28] based on molecular docking could improve the efficiency and save the cost of drug discovery. There have been several successful cases that use grid computing technology to perform virtual screening [29]–[35].

Molecular docking can only provide static interaction between the ligand and protein. It cannot provide enough information about the details of dynamic interaction process of protein and ligand. Molecular dynamics (MD) simulations have been proved to be very useful to explore the dynamical interaction between protein and ligand. MD simulations have been successfully used to study the interaction mechanism of the active and inactive states of β_2_ adrenergic receptor (β_2_AR) in complex with different ligands [36]. Based on MD simulations and anisotropic network model, the ligands were considered as “computational probe” to distinguish the different conformations of β_2_AR [37]. Free energy calculations of agonist, antagonist and inverse agonist of β_2_AR indicated that different ligands had significant difference of binding affinity and free energy [38]. MD simulations also proved that the crystal waters in the pocket of β_2_AR could form hydrogen bonds network to stabilize the agonist-receptor interaction [39]. The long unbiased MD simulations showed that there were two energetic barriers when the ligands entered into the pocket of β_2_AR [40]. At the same time, the dissociation pathway between ligands and β_2_AR was identified in the two secondary binding pockets in the extracellular part of β_2_AR [41]. MD simulations of β_2_AR in complex with Gs protein, which showed the interaction between the agonist BI-167107 and β_2_AR, supplied an activation mechanism of β_2_AR [42]. β_2_AR in complex with the inverse agonist, antagonist and agonist showed only the inverse agonist could induce the motion of Gαs and Gβγ domain by changing the conformation of β_2_AR [43].

In this work, we developed a new virtual screening program called MolGridCal based on grid computing. Furthermore, binding modes of possible ligands were further studied by combining molecular docking, MD simulations and free energy calculations. To test the use of the developed program and strategy, β_2_AR was selected as the model target. β_2_AR is distributed in the smooth muscle round the human body and plays an important role on the asthma and Alzheimer's disease (AD) [44]–[46]. The ligands of β_2_AR could regulate the smooth muscle relaxation, the vasodilation of muscle and liver, the dilation of bronchial passages, the relaxation of uterine muscle, insulin release and treat asthma and pulmonary disease [47]–[54]. In this work, MolGridCal was used to screen the ligands of β_2_AR from small molecule database. The screened ligands were refined by more accurate docking and scoring methods. The dynamic interaction mechanism between ligands and β_2_AR was further investigated by MD simulations and free energy calculations. Our strategy of virtual screening could not only extract the confirmed ligands from the small-database successfully, but also provided a more useful and accurate way to screen a large number of small molecule database.

## Materials and Methods

### Protein and Ligand Preparation

To screen the ligands of β_2_AR, the active conformation of β_2_AR was extracted from PDB database (PDB code: 3SN6 [55]). The Gs protein and ligand were removed in molecular docking. AutoDockTools [18], [56] was employed to add the Gasteiger charges and polar hydrogen on β_2_AR. The grid box in the pocket of β_2_AR was set to 30 Å×30 Å×30 Å around the position of ligand of β_2_AR. Autodock VINA [16] was chosen to screen the ligands of β_2_AR.

Here, 50,000 drug-like molecules with ionization states at pH 7 were selected from ZINC database [22], [57]. These 50,000 drug like molecules together with the agonist BI-167107 [55], neutral antagonist alprenolol [58] and inverse agonist ICI 118,551 [58] formed a small molecule database for virtual screening in grid computing environment.

### Grid Computing

To perform virtual screening in the grid computing environment, a program MolGridCal on basis of JPPF environment was designed in this work. The source codes of MolGridCal were freely available at http://molgridcal.codeplex.com. In the MolGridCal environment, we could use two modes of load-balancing of JPPF (http://www.jppf.org): one was “manual” mode which sent the fixed number of tasks to the each node; the other was “autotuned” algorithm which used the adaptive heuristic algorithm of Monte Carlo algorithm to transfer tasks to the calculation nodes. The total running time of MolGridCal was calculated as equation 1:
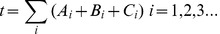
(1)
*A_i_* was the time of initial program to download ligand files from the FTP server. *B_i_* represented the time to run molecular docking. *C_i_* was the time of current tasks to end to upload the docking results.

The parameters of MolGridCal can be set easily. Firstly, it needs to know the IP address of FTP server, the small molecule database and uploading directory on FTP server. Secondly, it needs to specify which program of molecular docking will be called. In order to save the memory of computers, we recommend about 50,000 molecules per virtual screening in one folder. Of course, if the computers have enough memory for the cache store, the number of molecules for per virtual screening can be raised in one folder. If more than ten million small molecules are needed for virtual screening, we can use a program called VSBath in MolGridCal software package. VSBath can read the folder name to carry out the grid computing task one by one. The memory of computers will be able to release in time. The final network in our experiment contained 40 computers as computation nodes and 1 computer as the server node.

### Refinement by Accurate Molecular Docking

The virtual screening was carried out based on the crystal structure of β_2_AR using MolGridCal and Autodock VINA. In order to obtain more reliable and accurate virtual screening results, several other docking software including LibDock [59], CDOCKER [60] and Flexible Docking modules of Discovery Studio 2.5 (DS2.5) were employed. In Libdock, the ligands were docked into the protein based on the polar and nonpolar hotspot of the features of protein active sites. In this experiment, 100 grids were generated in the region of radius of 8 Å around the agonist-bound sites of β_2_AR. CHARMM force field [61] was employed for energy minimization. CDOCKER was a semi-flexible docking program on basis of molecular dynamics. High temperature molecular dynamics simulation was used to search the flexible conformation of ligands. Simulated annealing method was employed to optimize the conformation on the active site of receptor. The heating target temperature was set to 700 K, and the heating steps were assigned to 2000. In DS Flexible Docking, the side chain clusters were generated using ChiFlex method. DS Flexible Docking based on CDOCKER method was used in simulated annealing and energy minimization. In the DS Flexible Docking, the residues of radius of 4 Å around BI-167107 in the β_2_AR were chosen as the flexible residues. The same parameters with CDOCKER method was used in the energy minimization and simulated annealing.

### Molecular Dynamics Simulations

The crystal structure of β_2_AR was obtained from PDB database (PDB code: 3SN6 [55]). The T4 lysozyme, nanobody (Nb35) and BI-167107 were deleted from the crystal structure of β_2_AR. The lack loop sequence (FHVQNLSQVEQDGRTGHGLRRSSKF) of TM5 and TM6 was built by MODELLER program [62]. The obtained loop region was further optimized by loop model algorithm [62]. β_2_AR was then modeled in complex with the agonist BI-167107, antagonist alprenolol and inverse agonist ICI 118,551, respectively. The explicit membrane around the transmembrane region was constructed using the 1-palmitoyl-2-oleoyl-sn-glycero-3-phosphocholine (POPC) lipids. The sizes of membrane were 120 Å×120 Å. The complex of β_2_AR and Gs (β_2_AR-Gs) protein was immersed into TIP3P water [63] box along Z direction. To get the neutral system, seven sodium ions were added into the water box. The entire system, whose dimension was 120 Å×120 Å×150 Å, contained the lipids, water, ions, ligands, α-helix domain, Gαs, Gβγ and β_2_AR. The atomic number of final system was about 200,010 per periodic cell. The CHARMM force field parameters of BI-167107, alprenolol and ICI 118,551 were modeled using VMD Paratool Plugin v1.2 [64], [65] and Gaussian 98 Revision A.9 [66]. The geometry optimization and single point calculation were both performed at the theory of RHF/6–31G* level and tight SCF convergence criteria.

MD simulations were performed on the β_2_AR in complex with different ligands. The lipid tail was minimized for 100 ps and equilibrated for 1000 ps at the constant temperature of 300 K and constant pressure of 1 bar firstly. By constraining the protein and ligand, the studied systems were further minimized for 100 ps based on the conjugate gradient method and equilibrated for 1000 ps. Then the whole systems were equilibrated freely for 5 ns. At last, 10 ns MD simulations were run. All MD simulations were performed using NAMD (version 2.9b3) [67] with CHARMM 27 force field [61] in the periodically infinite lipid and explicit solvent. The particle-mesh Ewald (PME) [68] method was used to calculate the electrostatics with a 12 Å nonbonded cutoff. The constant temperature of 300 K and pressure of 1 bar employed the langevin thermostat and langevin barostat [69] method, respectively. Time step was set to 2 fs. The trajectory frames were save every 1 ps for analysis. All MD simulations were carried out on 12 cores of an array of two 2.66-GHz Intel Xeon 5650 processors and 4 pieces of NVDIA Tesla C 2050 GPU computing processors. To study the antagonist and inverse agonist in their native crystal receptor, we built two MD simulation systems based on the crystal structures of β_2_AR in complex with antagonist alprenolol (PDB ID: 3NY8) and inverse agonist ICI 118,551 (PDB ID: 3NYA). The size of POPC membrane was set to 80 Å×80 Å. The final dimension of system boxes were 80 Å×80 Å×100 Å. MD simulations on these two complexes were ran using the same parameters with above agonist-bound β_2_AR.

### Free Energy Calculations

Adaptive biasing force (ABF) method [70]–[72] can provide details about the free energy of dissociation between the ligand and protein using the biasing force which could offset the local barriers effectively. Here, the reaction coordinate (RC) was projected onto the Z direction. The free energy ΔG along the Z axis was defined as equation 2:
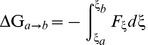
(2)
*F_ξ_* represented the biasing force. The center of A119, F208, T283 and N318 was selected as the reference point. Because the length of Z direction between the ligand and water box was about 25 Å, the ligand had enough space to get out of the pocket of β_2_AR with respect to reference point. Two no-overlapping widows were divided for free energy calculation. 10 ns ABF simulations were performed on each window. The bin width of ABF simulation was 0.2 Å. The boundary potentials were set to a force constant of 50 kcal/mol/Å^2^. The biasing force carried out every 500 samples in a bin. All the ABF simulations were realized by NAMD (version 2.9b3) [67].

## Results and Discussion

### The Algorithm of MolGridCal

**Figure 1:**
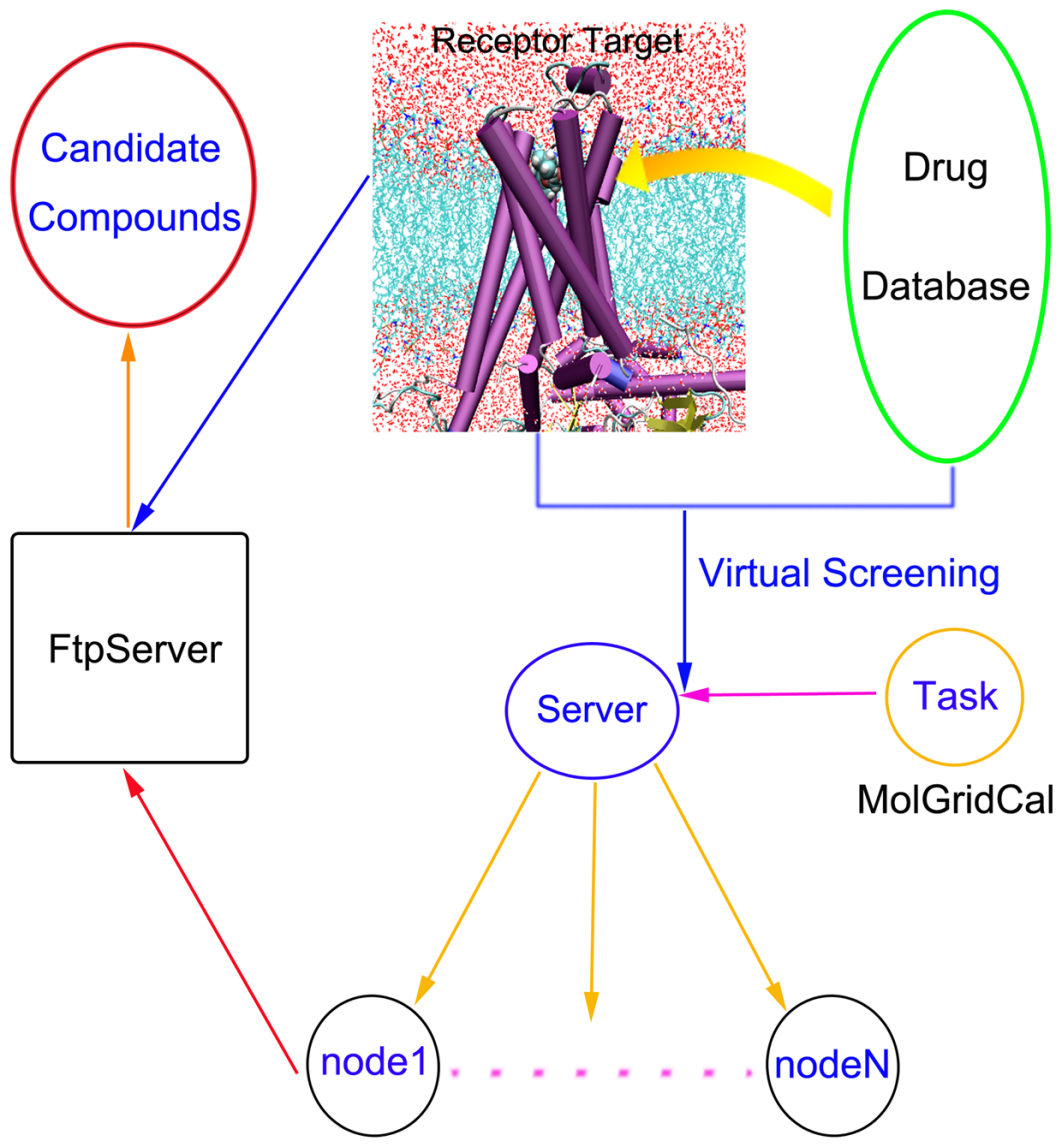
The flowchart of virtual screening based on grid computing. MolGridCal can firstly submit the works into the server. Then the server distributed works into different nodes. The finished works would be gathered to find the candidate compounds.

The MolGridCal program was designed by using grid computing based on the framework of JPPF. MolGridCal could package small molecule database, IP address of FTP server, docking program and corresponding folder automatically (Figure 1). The server node would distribute these tasks to idle connected nodes. The nodes could adopt the flexible options for virtual screening. The number of used cores of computers could be set in the configure file of Autodock VINA. If there was any action of mouse and keyboard, the tasks of MolGridCal would be terminated until the action stopped. At the same time, the terminated work was sent to the idle computers. MolGridCal would send all the docking results to the FTP server automatically. The collected results could be ranked according to the docked binding affinity.

**Figure 2:**
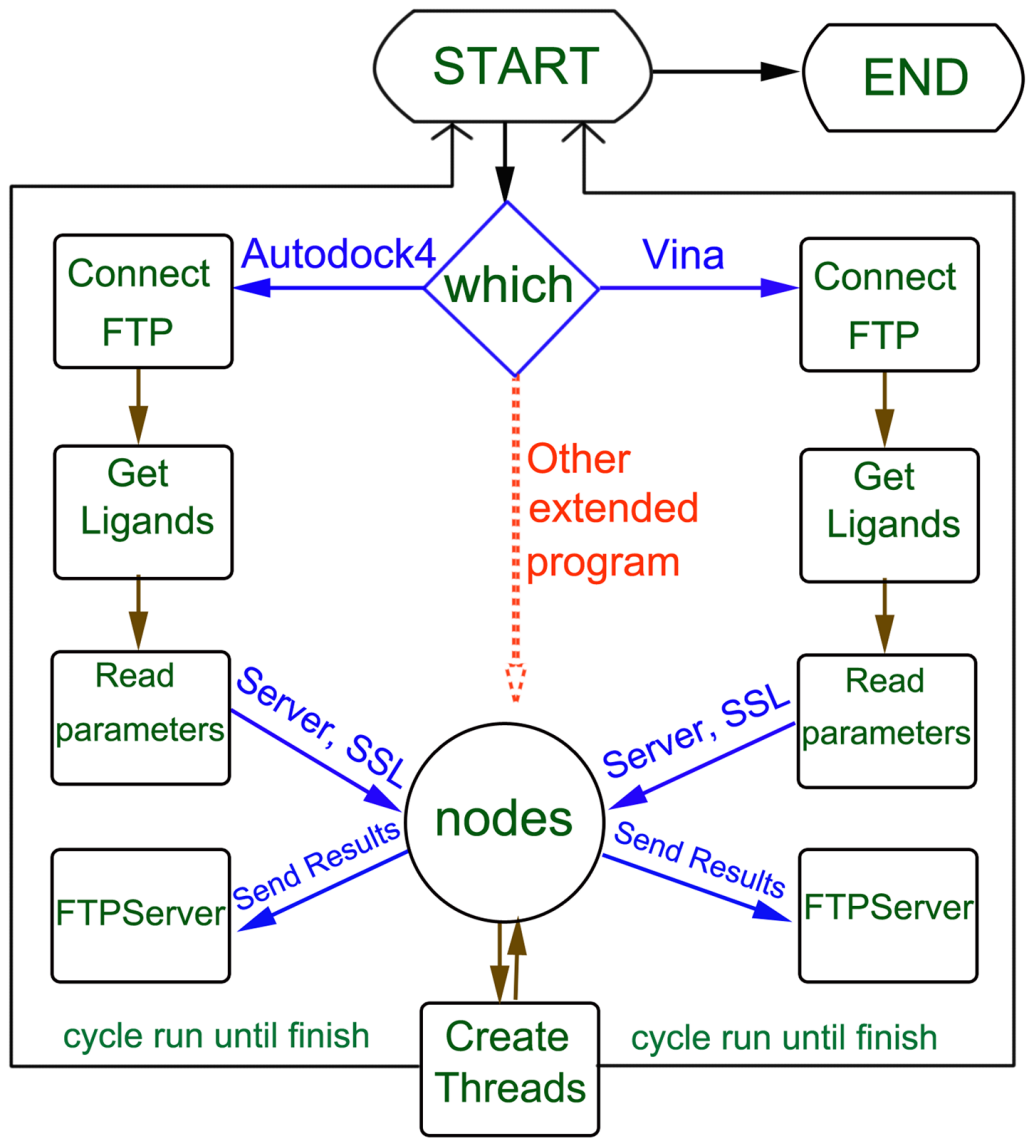
The implementation process about of MolGridCal.

The flowchart of MolGridCal was shown in Figure 2. Firstly, the docking program should be chosen for grid computing. At the same time, the messages of IP address, username, password, download and upload directory were bundled as a package. MolGridCal would guide the nodes to connect the FTP severer using the bundle of verified message and to download the ligands into the local machine automatically. To make sure the transferring security of the message, Secure Socket Layer (SSL) was employed. All the process of connection, upload and download need certificate validation. To guarantee the authority of submission in the grid computing network, MolGirdCal used the unique certificate to connect the server. Only the corrected request certificate could exchange the message. The symmetric algorithm was used for server, nodes and MolGridCal. When the message was closed, the SSL finished the “handshake” between the server and clients (Figure S1). The SSL could make sure the exchange message to be secure and reliable. Once the tasks were sent to the nodes, the nodes would create threads to perform molecular docking. To save the memory of computer, the thread was ended instantly once the molecular docking task was finished. The final docking results would be uploaded to the FTP server automatically. MolGridCal would execute the grid computing until all tasks were finished (see Figure 2).

### Virtual Screening Based on MolGridCal

Many factors might affect the efficiency of grid computing. The network delay, time of re-allocated tasks and configure of computers were the main factors to postpone the tasks of MolGridCal. The manual and autotuned modes provided by JPPF, were used for tasks allocation. These two modes could send the defined number of docking tasks to the nodes by modifying the size parameter of server. If manual algorithm was applied, the tasks would be sent to nodes batch by batch regularly. If the autotuned mode was chosen for MolGridCal, the tasks would pick the suitable way to send the tasks to the nodes using the adaptive heuristic algorithm of Monte Carlo method.

**Figure 3:**
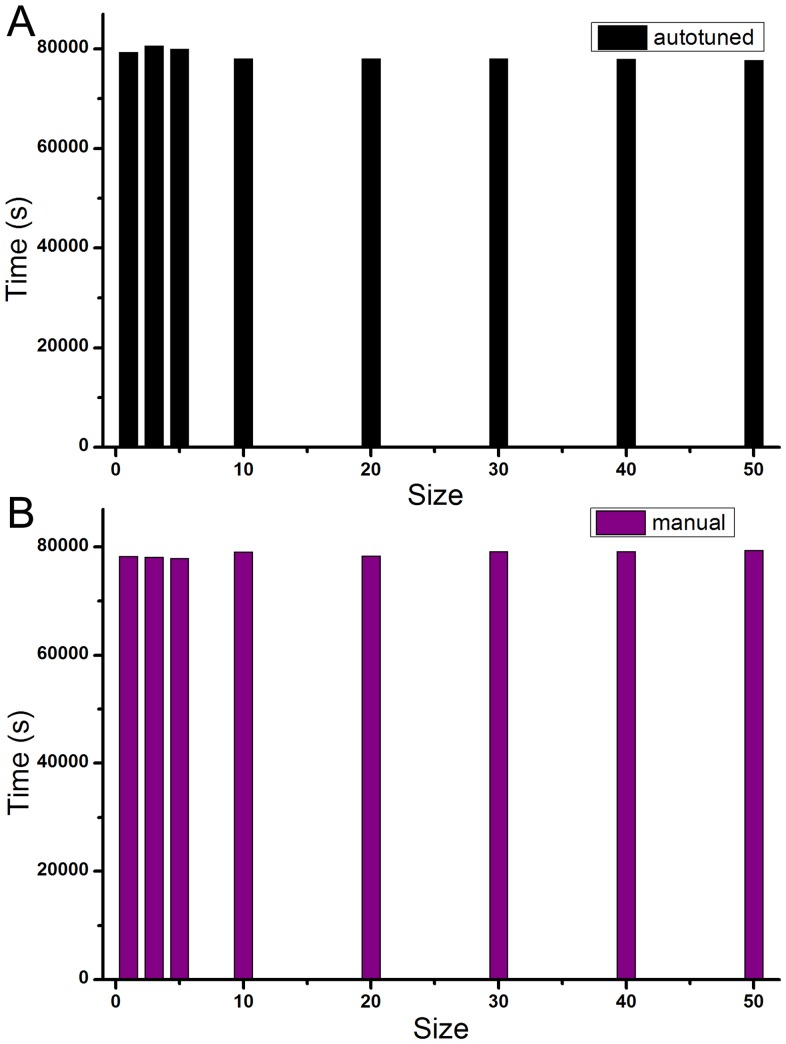
Total running time versus number of tasks. (A) Running time using autotuned algorithm. (B) Running time using manual algorithm. The size represented the number of tasks.

**Figure 4:**
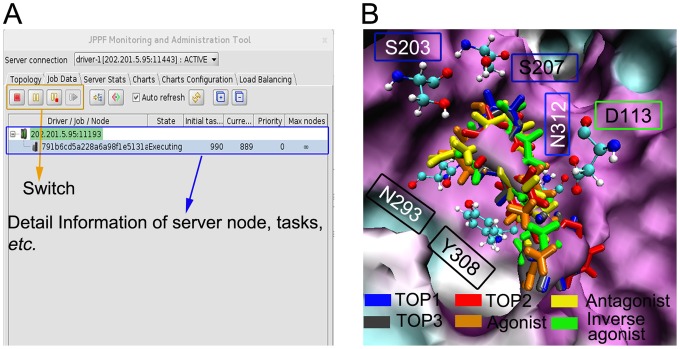
The GUI of MolGridCal and the binding pocket of β_2_AR. (A) The displayed information of GUI for monitoring MolGridCal. (B) The top three compounds, agonist, antagonist, inverse agonist bound to the pockets of β_2_AR. Different ligands were represented by different colors. TOP1,2,3, agonist BI-167107, antagonist alprenolol and inverse agonist ICI 118,551 were colored in blue, red, gray, orange, yellow and green, respectively.

As shown in equation 1, the differences of total running time were mainly determined by the download, upload and molecular docking time. If the network was slow, the bottleneck of time was due to the process of download and upload. In contrast, if the computers of nodes ran slowly, a bulk of tasks would be postponed on these computers. On basis of these factors, a grid computing network composed by 40 computers as computation nodes and 1 computer as server was used in virtual screening. The number of test tasks was set 1, 3, 5, 10, 20, 30, 40 and 50 to test the speed of this grid computing network, respectively (Figure 3). Figure 3A and 3B illustrated the total time with respect to the numbers of tasks which were sent to the nodes using the manual and autotuned modes, respectively. In the autotuned mode, the total running time would keep stable with the increased task size. The tasks were distributed into the nodes using adaptive heuristic algorithm randomly, so the entire running time had no large fluctuation. For the manual mode, it had the same situation. The final running time was mainly determined by the computer speed in the nodes. The whole virtual screening time for the 50,000 molecules was about 22 hours in the above grid computing environment (Figure 3). JPPF also can supply Graphical User Interface (GUI) to operate the computation nodes (Figure 4A). The GUI can not only give the information about the tasks states of MolGridCal, but also can restart, suspend and terminate the tasks easily.

### Refinement of Results from Virtual Screening

The virtual screening results from MolGridCal and Autodock VINA were collected from FTP server, and ranked according to the docking score. The agonist BI-167107 fell into top 2% of virtual screening results, while the antagonist alprenolol and inverse agonist ICI 118,551 were ranked out of top 42% and 33% of virtual screening results. To further refine the screened results, LibDock and CDOCKER were chosen to perform accurate molecular docking. The above agonist, antagonist and inverse agonist as well as molecules of TOP1 (ZINC ID: 00155747), TOP2 (ZINC ID: 00298339) and TOP3 (ZINC ID: 00155744) ranked in the top 3 from the small molecule database were chosen for further analysis (Table 1). The structures of TOP1-3 were shown in Figure S2. LibDock score gave different ranking from AutoDock Vina. The agonist BI-167107 gave the highest docking score and was ranked in top 1.The ranking order of TOP2, TOP3 and alprenolol was changed. When CDOCKER was employed, the ranking results changed again. The agonist BI-167107, antagonist alprenolol and inverse agonist ICI 118,551 were ranked in top 3, while the TOP1, TOP2 and TOP3 (TOP1,2,3) were ranked behind the inverse agonist ICI 118,551. It indicated that the CDOCKER could find the potential ligands (agonist, antagonist and inverse agonist). The results of DS Flexible Docking had the same order of docking results with CDOCKER. It showed that the agonist BI-167107 was ranked as the best one. According to the experiment results in the references [73]–[75], the p*K*_i_(s) of ICI 118,551 and alprenolol were 9.2 and 9.0, respectively. The agonist had higher binding affinity than inverse agonist in the pocket of β_2_AR [76], [77].

**Table 1:** The docking scores of different ligands using Autodock VINA, LibDock, CDOCKER and Flexible Docking.

	AutoDock VINA (kcal/mol)	LibDock Score	CDOCKER (kcal/mol)	Flexible Docking (kcal/mol)
BI-167107	−10.6	155.846	−50.842	−51.008
Alprenolol	−7.2	111.429	−34.728	−38.276
ICI 118,551	−8.3	110.088	−21.709	−28.418
Top 1	−12.0	129.346	−17.769	−23.556
Top 2	−11.8	125.073	−10.599	−14.274
Top 3	−11.6	128.991	−9.783	−14.167

**Figure 5:**
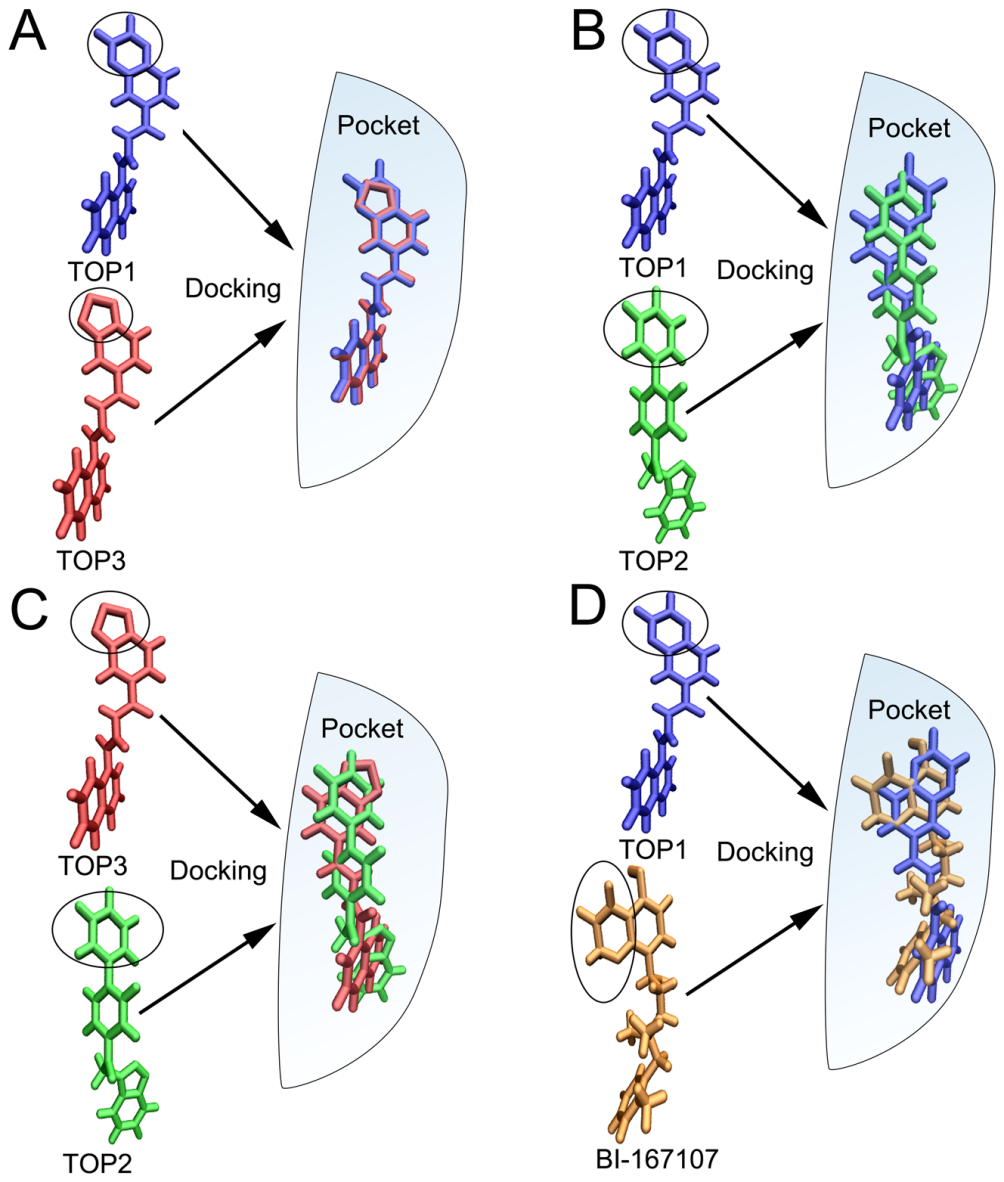
Analysis of conformation of different ligands. Delineating conformational differences between (A) TOP1-TOP3, (B) TOP1-TOP2, (C) TOP2-TOP3, (D) TOP1-BI-167107 in the pocket of β_2_AR. The black oval showed the key atoms for the binding pocket of β_2_AR.

**Figure 6:**
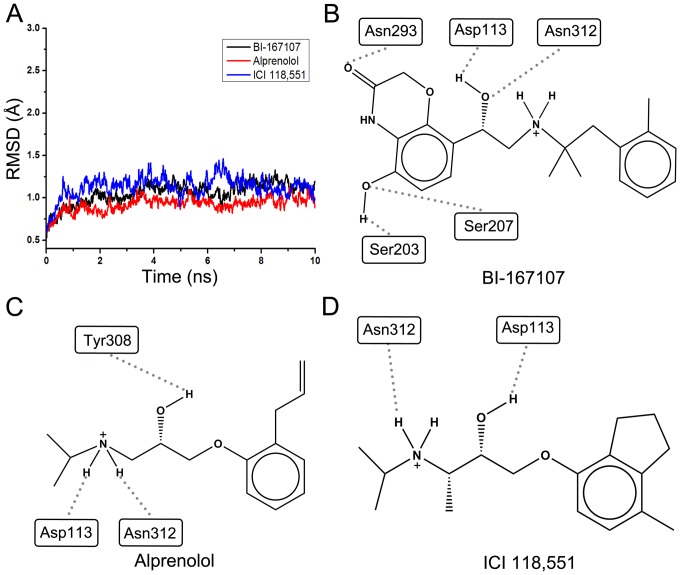
RMSD and interactions between ligands and residues of β_2_AR. (A) RMSD of the backbone atoms of β_2_AR in complex with BI-167107, alprenolol and ICI 118,551 during MD simulations. (B–D) The hydrogen bonds interaction between the residues of β_2_AR and three ligands: BI-167107, alprenolol, ICI 118,551, respectively.

Figure 4B illustrated the binding mode of agonist, antagonist and inverse agonist in β_2_AR. All the ligands were surrounded by D113, S203, S207, N293, Y308 and N312 in the binding pocket of β_2_AR. To further study the binding mode of different ligands, the top three small molecules and agonist BI-167107 were chosen (see Figure 5). Molecule TOP1 had the similar structure with the TOP3. Molecules TOP1 and TOP3 could overlap each other well in the binding pocket of β_2_AR. Molecule TOP1 had a higher binding affinity than TOP3. The difference was that TOP1 had a quinoxaline group. At the same position, TOP3 contained a benzothiadiazole group (black oval of Figure 5A). By comparing the structures of TOP1 and TOP2, it could be seen that there was a phenyl group in TOP2 at the position of quinoxaline group of TOP1 (Figure 5B). In Figure 5C, it could be seen obviously that the benzothiadiazole group of TOP3 superimposed with the phenyl group of TOP2. According to the binding mode, it could be inferred that the quinoxaline group had more favorable binding with β_2_AR. By comparing TOP1 and BI-167107 (Figure 5D), it could be seen that the benzoxazine group of BI-167107 had different position with the quinoxaline group of TOP1. The position of benzoxazine group of BI-16710 could form hydrogen bonds with ASN293 (Figure 6B). The pharmacophore model of β_2_AR agonists was also built by Schrödinger Suite 2009 software (Table S1 and Figure S3). It also showed that the benzoxazine group of BI-167107 had the common hydrogen bond donor with other agonists.

### Molecular Dynamics Simulations and Free Energy Calculations

Although molecular docking could provide information about the interaction of ligand with the key residues in the active site of the protein, it could not give the fully dynamical interaction between the ligand and target. Molecular dynamics (MD) simulations could provide more information about the dynamical interaction between the active sites of protein and ligands along the simulation time. Besides, free energy calculation could give more accurate evaluation of the binding ability of ligands based on the MD simulations. MD simulations combined with binding free energy calculation were used to refine the results of virtual screening and to give more deeply understanding of interaction mechanism between the obtained candidate ligands and protein. ABF method was used to calculate the binding free energy of the ligands to β_2_AR. The agonist BI-167107, antagonist alprenolol and inverse agonist ICI 118,551 were chosen for the further MD simulations and free energy calculations. β_2_AR in complex with agonist, antagonist and inverse agonist reached equilibrium over 10 ns MD simulations (see Figure 6A). To make sure whether the membrane keeps stable, MEMBPLUGIN [78] was employed to measure membrane thickness during MD simulations. The results showed the membrane also reached equilibrium phase over 10 ns MD simulations (see Figure S7). MD simulation results showed that water molecules could form dynamical hydrogen bond networks to interact with the residues of β_2_AR (see Figure S4). This hydrogen bond network played an important role to stabilize conformation of β_2_AR during the MD simulations [79]–[81]. Figure 6B showed the formed hydrogen bonds between the agonist BI-167107 and residues Asp113, Ser203, Ser207, Asn293 and Asn312 of β_2_AR. Figure 6C illustrated Asp113, Tyr308 and Asn312 formed three hydrogen bonds with antagonist alprenolol. Figure 6D showed only Asp113 and Asn312 formed hydrogen bonds with inverse agonist ICI 118,551. To investigate the interaction between the residues of β_2_AR and different ligands, the number of hydrogen bonds of different ligands and β_2_AR were monitored during MD simulations [82], [83]. In addition, to validate the binding modes of the antagonist alprenolol and inverse agonist ICI 118,551 in their native crystal structures of β_2_AR, MD simulations were performed on two built inactive states of β_2_AR, respectively. As shown in Figure S5, two systems reached equilibrium phase over 10 ns MD simulations. Figure S6 showed that the inverse agonist ICI 118,551 mainly formed the hydrogen bonds with Asp113 and Asn312 except Tyr308, while the antagonist alprenolol had high hydrogen bonds occupancy with Asp113 and Asn312.

**Figure 7:**
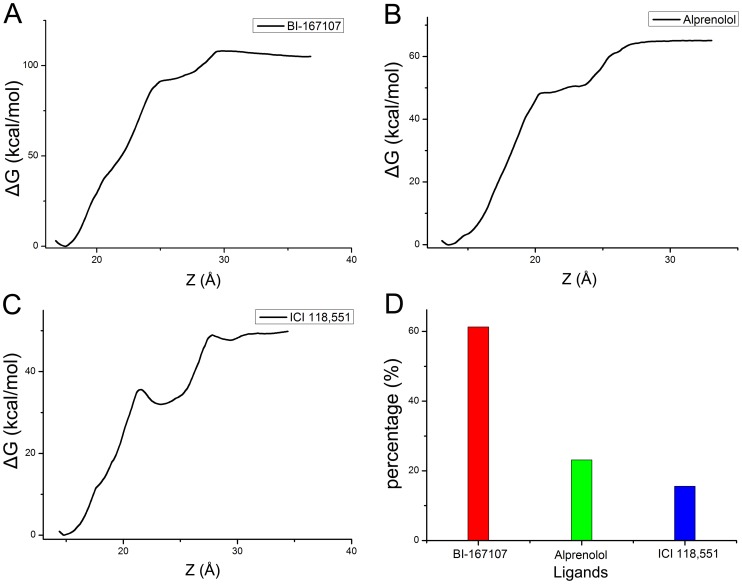
Free energy of ligands and hydrogen bonds percentage. (A–C) Free energy profiles were delineated when the BI-167107, alprenolol and ICI 118,551 got out of the pocket of β_2_AR along the Z axis direction, respectively. (D) The hydrogen bonds percentages of BI-167107, alprenolol and ICI 118,551.

By stretching the ligands out of the binding pocket, ABF simulations could give information about the interaction energy change during this process (Figure 7). Figure 7A and Movie S1 was the free energy corresponding to dynamically stretching process of the agonist BI-167107. The agonist BI-167107 needed to overcome about 105 kcal/mol energy barriers to get out of β_2_AR. Figure 7B and Movie S2 showed the free energy along Z axis and the animation about the interaction between alprenolol and β_2_AR. The antagonist alprenolol needed to overcome about 65 kcal/mol energy barriers to get out of the pocket of β_2_AR. Figure 7C and Movie S3 illustrated the free energy and stretching process of the inverse agonist ICI 118,551. ICI 118,551 needed to overcome about 49 kcal/mol energy along Z axis. The difference of free energy along Z axis direction further proved the agonist had the strongest binding affinity to β_2_AR. Furthermore, the binding mode analysis based on the complexes obtained from MD simulations showed the hydrogen bonds interaction might contribute to the different binding ability of three ligands. Figure 7D illustrated the hydrogen bonds percentage of different ligands. The result further indicated that the BI-167107 could bind to the pocket of β_2_AR better than other ligands since the BI-167107 could form more hydrogen bonds along dissociation pathway of ligands. MD simulations and free energy calculations could provide more information about the dynamical interaction between ligands and protein.

## Conclusions

In this work, we designed MolGridCal program for virtual screening of ligands of β_2_AR using grid computing by combination use of molecular docking, MD simulations and free energy calculations. MolGridCal could send a serial of works into the nodes for computing automatically. The nodes could implement grid computing easily by using idle computer resource. The virtual screening strategy was further tested by using β_2_AR as a model target and 50,000 ligands as small molecule database. The results indicated that our virtual screening strategy could successfully find the agonist BI-167107 from the small molecule database. To further detail the interaction difference between the ligands and β_2_AR, MD simulations and free energy calculations were performed on the β_2_AR in complex with BI-167107, alprenolol and ICI 118,551. The MD simulations and free energy indicated the agonist BI-167107 had the highest free energy along reaction coordinate. This virtual screening strategy could also be applied to screen drug for other targets.

## Supporting Information

Figure S1 **The principle of message exchange by SSL.**
(TIF)

Figure S2 **The molecular structures of TOP1 (ZINC ID: 00155747), TOP2 (ZINC ID: 00298339) and TOP3 (ZINC ID: 00155744).**
(TIF)

Figure S3 **The pharmacophore model of agonists of β_2_AR.** The pharmacophore model was generated by the agonists in Table S1. A1: hydrogen bond acceptors, D5 and D7: hydrogen bond donors, R10: aromatic rings.
(TIF)

Figure S4 **The hydrogen bonds networks of water molecules in the pocket of β_2_AR-bound to BI-167107, alprenolol and ICI 118,551.** The orange color part represented the residues of β_2_AR. The blue and red lines were the hydrogen bonds.
(TIF)

Figure S5 **RMSD of the backbone atoms of β_2_AR in complex with alprenolol and ICI 118,551 versus simulation time.**
(TIF)

Figure S6 **The hydrogen bonds occupancy between β_2_AR and ICI 118,551, alprenolol.**
(TIF)

Figure S7 **The membrane thickness versus simulation time.**
(TIF)

Table S1 **The structures of β_2_AR agonists.**
(DOC)

Movie S1 **The escape process of agonist BI-167107 in the pocket of β_2_AR.**
(MPG)

Movie S2 **The escape process of antagonist alprenolol in the pocket of β_2_AR.**
(MPG)

Movie S3 **The escape process of inverse agonist ICI 118,551 in the pocket of β_2_AR.**
(MPG)
