# Supplementary figures and images for: Search for β2 Adrenergic Receptor Ligands by Virtual Screening via Grid Computing and Investigation of Binding Modes by Docking and Molecular Dynamics Simulations
Source: PLoS One. 2014 Sep 17;9(9):e107837. doi: 10.1371/journal.pone.0107837 (PMC4168136; doi:10.1371/journal.pone.0107837)

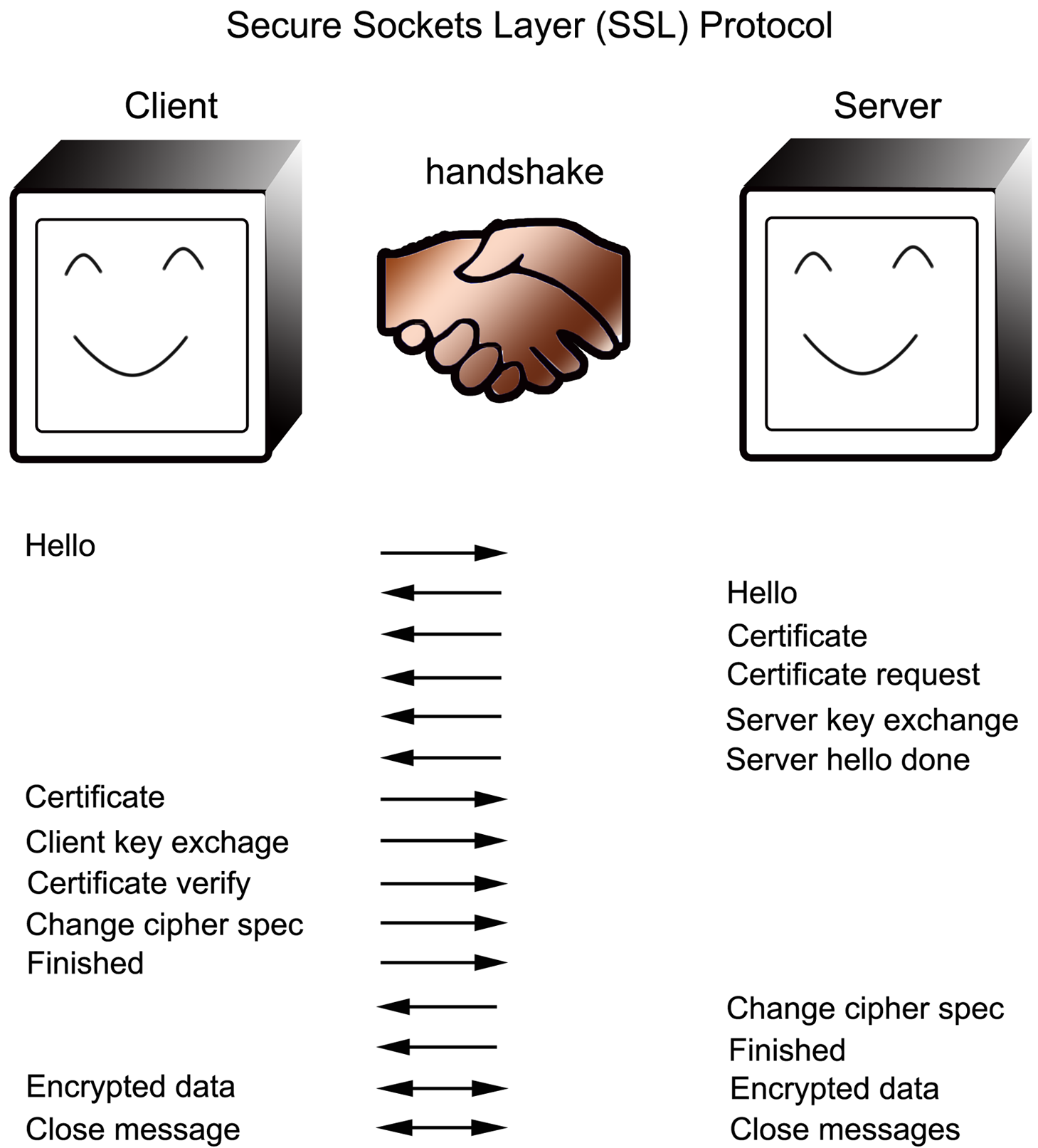

Supplement: Figure S1 — The principle of message exchange by SSL. (TIF) [file pone.0107837.s001.tif]

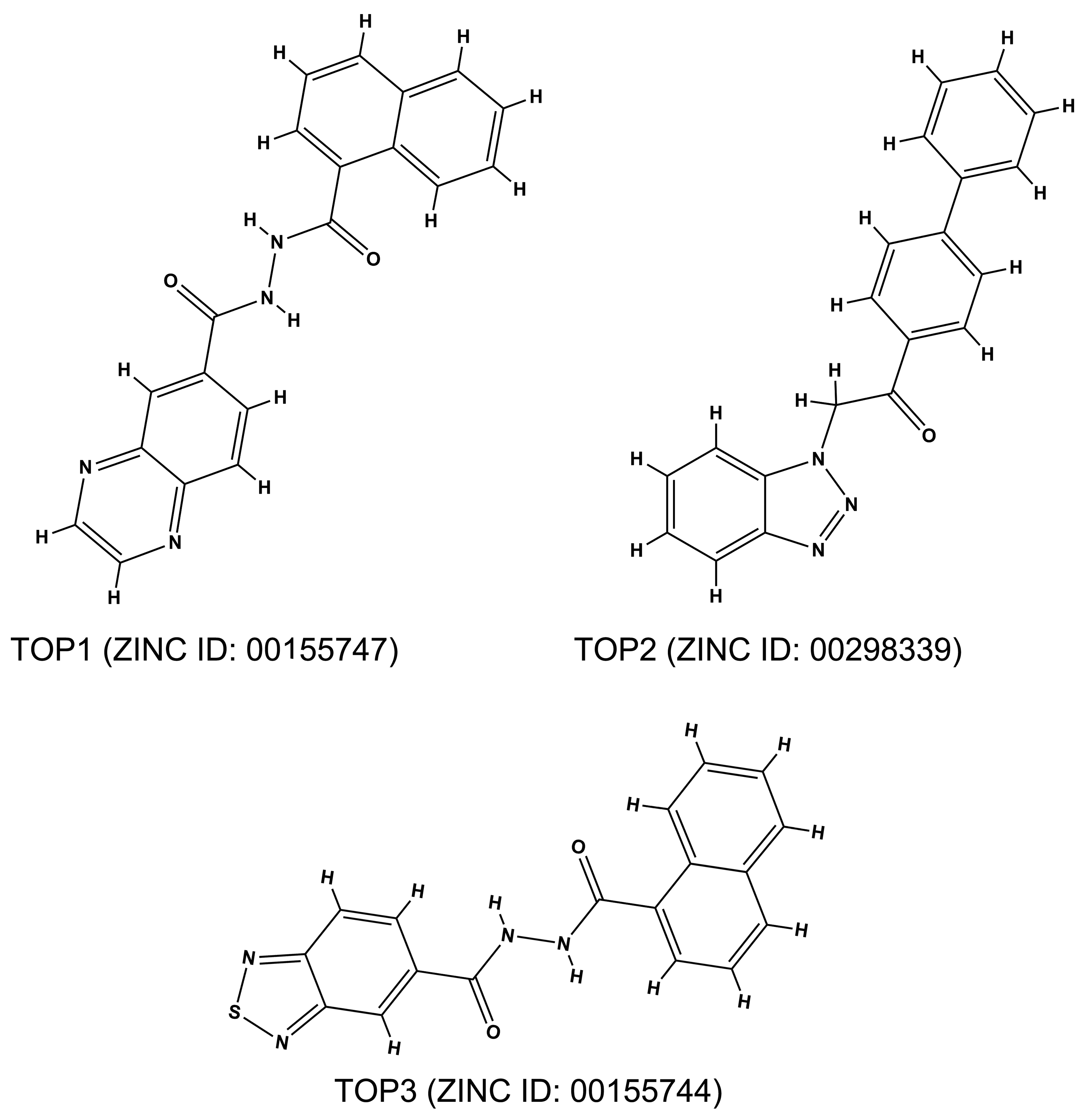

Supplement: Figure S2 — The molecular structures of TOP1 (ZINC ID: 00155747), TOP2 (ZINC ID: 00298339) and TOP3 (ZINC ID: 00155744). (TIF) [file pone.0107837.s002.tif]

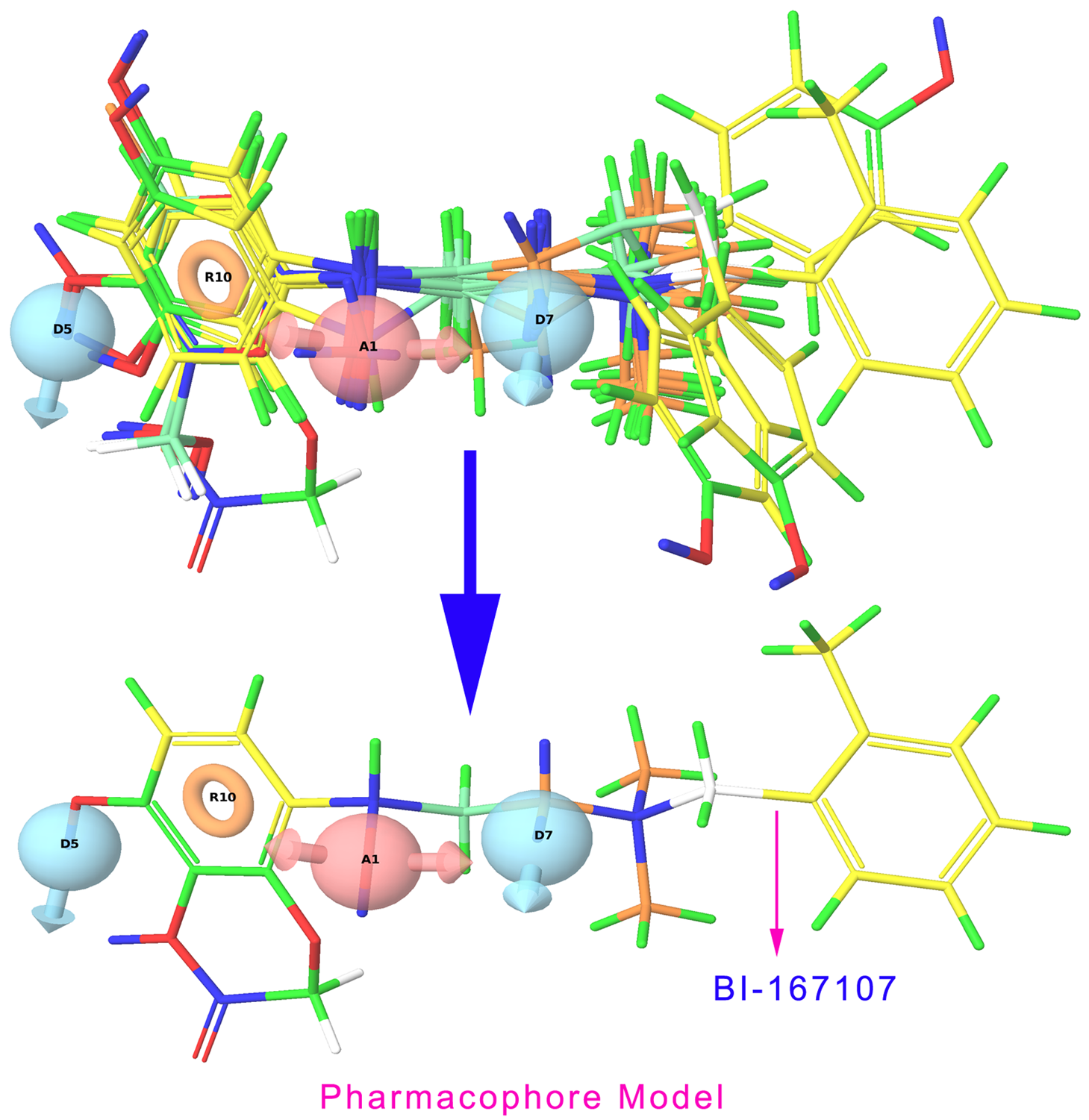

Supplement: Figure S3 — The pharmacophore model of agonists of β2AR. The pharmacophore model was generated by the agonists in Table S1. A1: hydrogen bond acceptors, D5 and D7: hydrogen bond donors, R10: aromatic rings. (TIF) [file pone.0107837.s003.tif]

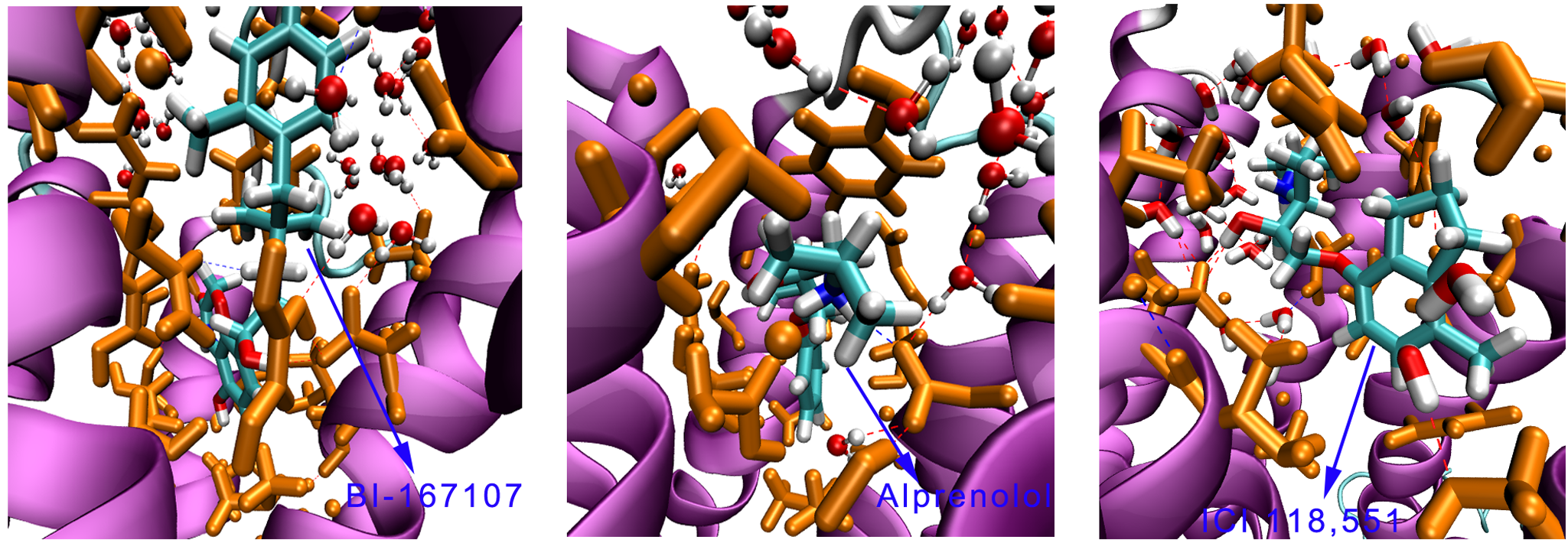

Supplement: Figure S4 — The hydrogen bonds networks of water molecules in the pocket of β2AR-bound to BI-167107, alprenolol and ICI 118,551. The orange color part represented the residues of β2AR. The blue and red lines were the hydrogen bonds. (TIF) [file pone.0107837.s004.tif]

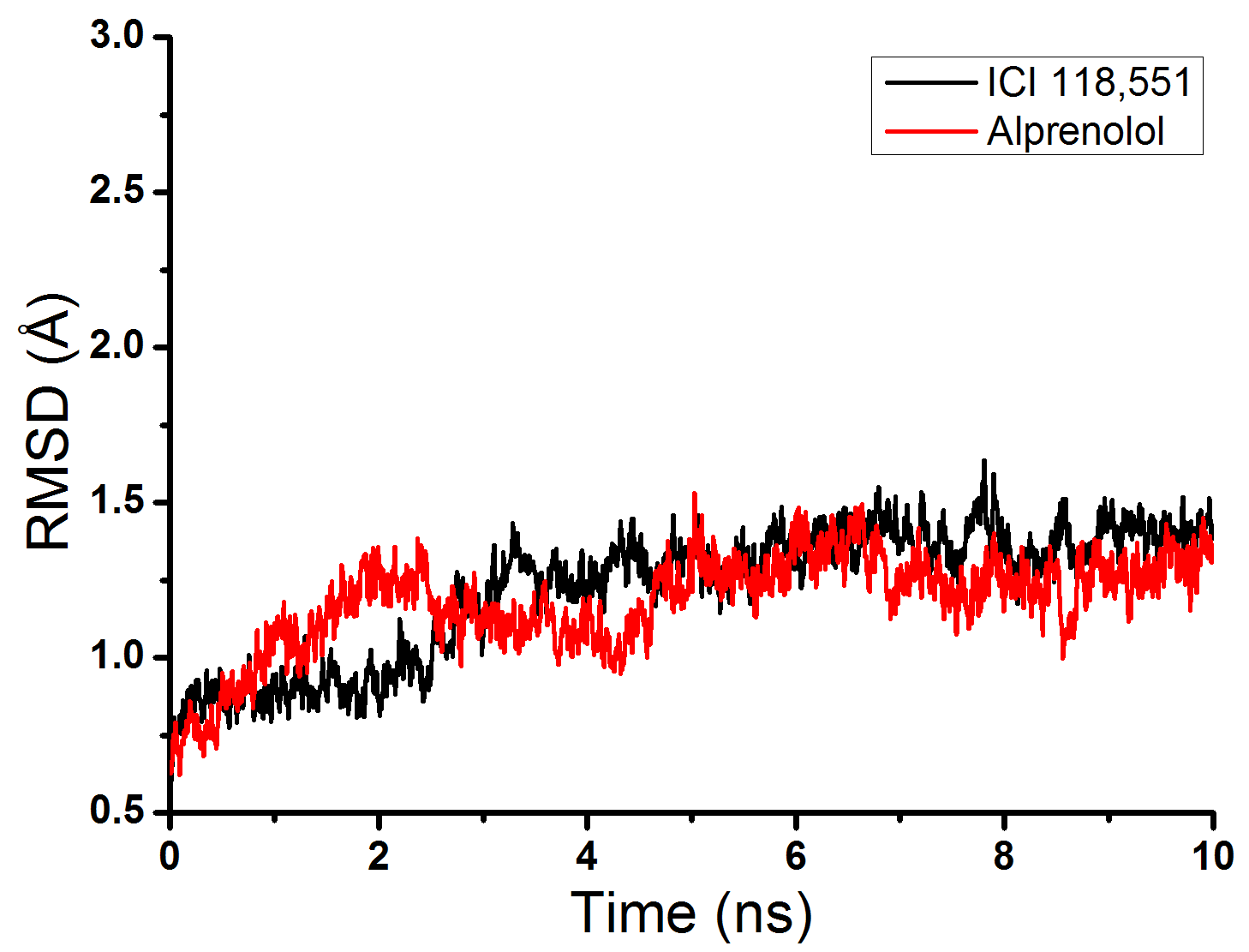

Supplement: Figure S5 — RMSD of the backbone atoms of β2AR in complex with alprenolol and ICI 118,551 versus simulation time. (TIF) [file pone.0107837.s005.tif]

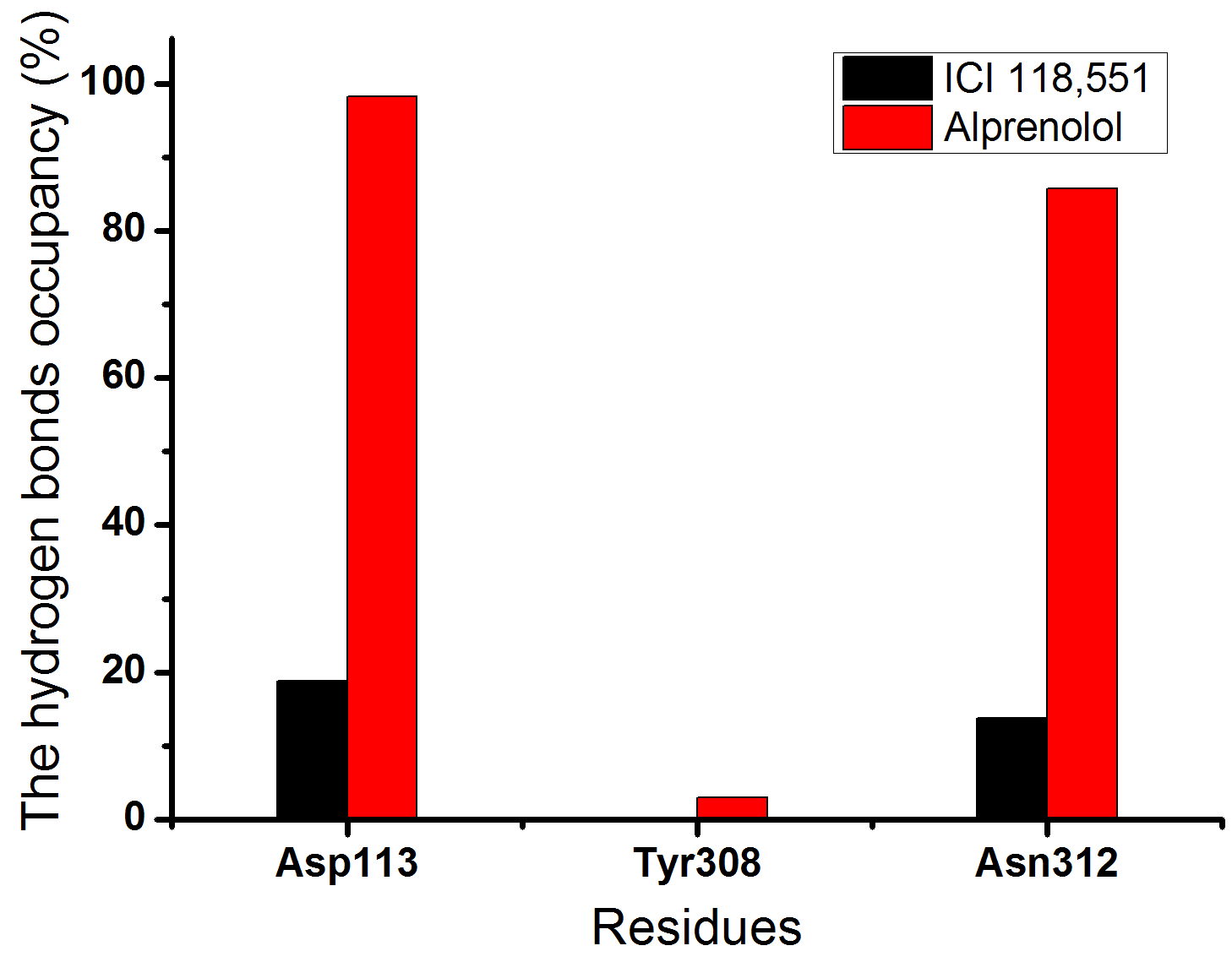

Supplement: Figure S6 — The hydrogen bonds occupancy between β2AR and ICI 118,551, alprenolol. (TIF) [file pone.0107837.s006.tif]

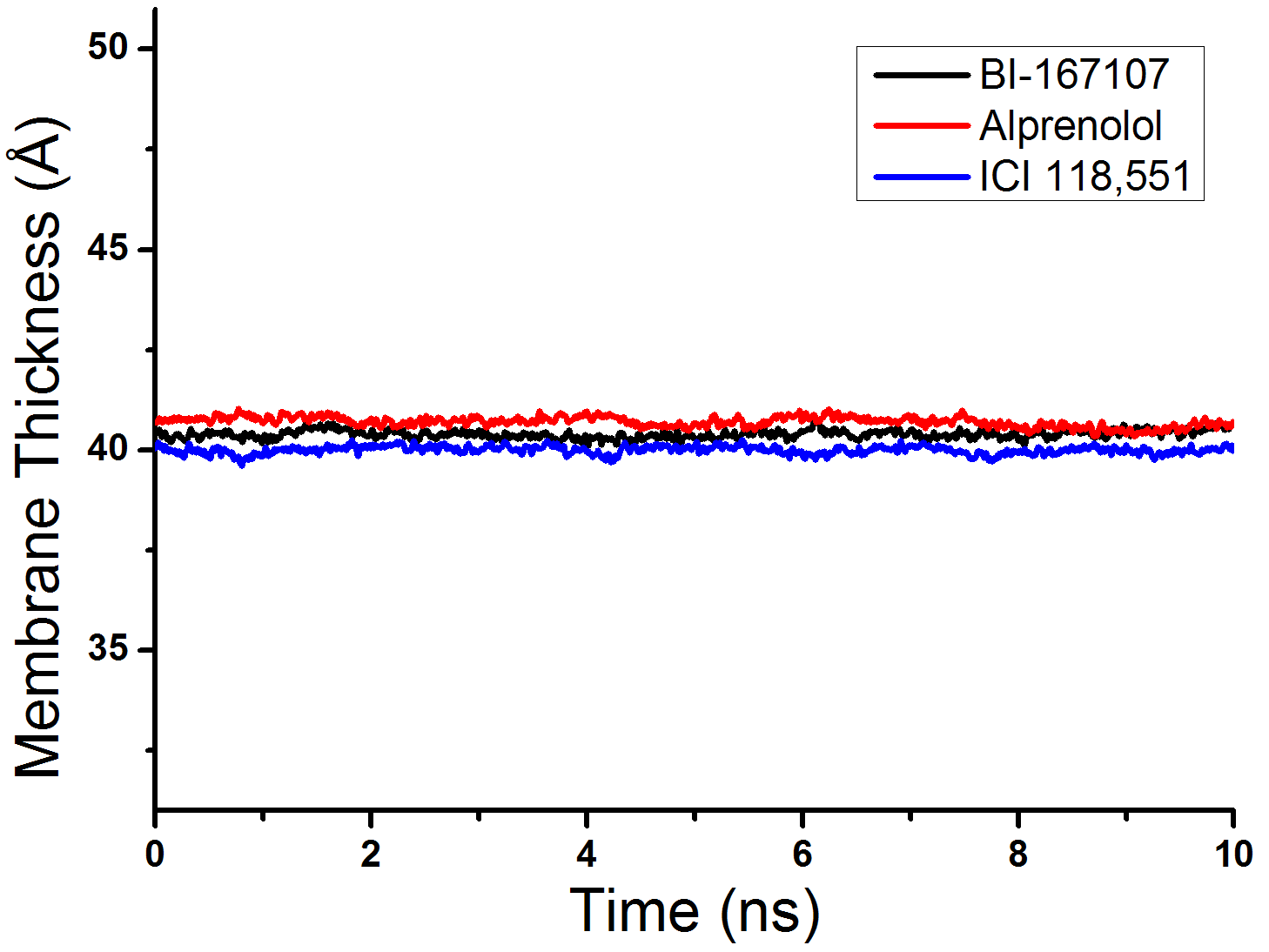

Supplement: Figure S7 — The membrane thickness versus simulation time. (TIF) [file pone.0107837.s007.tif]
