# Supplementary material for: Search for β2 Adrenergic Receptor Ligands by Virtual Screening via Grid Computing and Investigation of Binding Modes by Docking and Molecular Dynamics Simulations
Source: PLoS One. 2014 Sep 17;9(9):e107837. doi: 10.1371/journal.pone.0107837 (PMC4168136; doi:10.1371/journal.pone.0107837)
Supplement: Table S1 — The structures of β2AR agonists. (DOC) [file pone.0107837.s008.doc]

**Table S1.** The structures of β_2_AR agonists

| Agonists | Structures |
| --- | --- |
| Adrenaline | 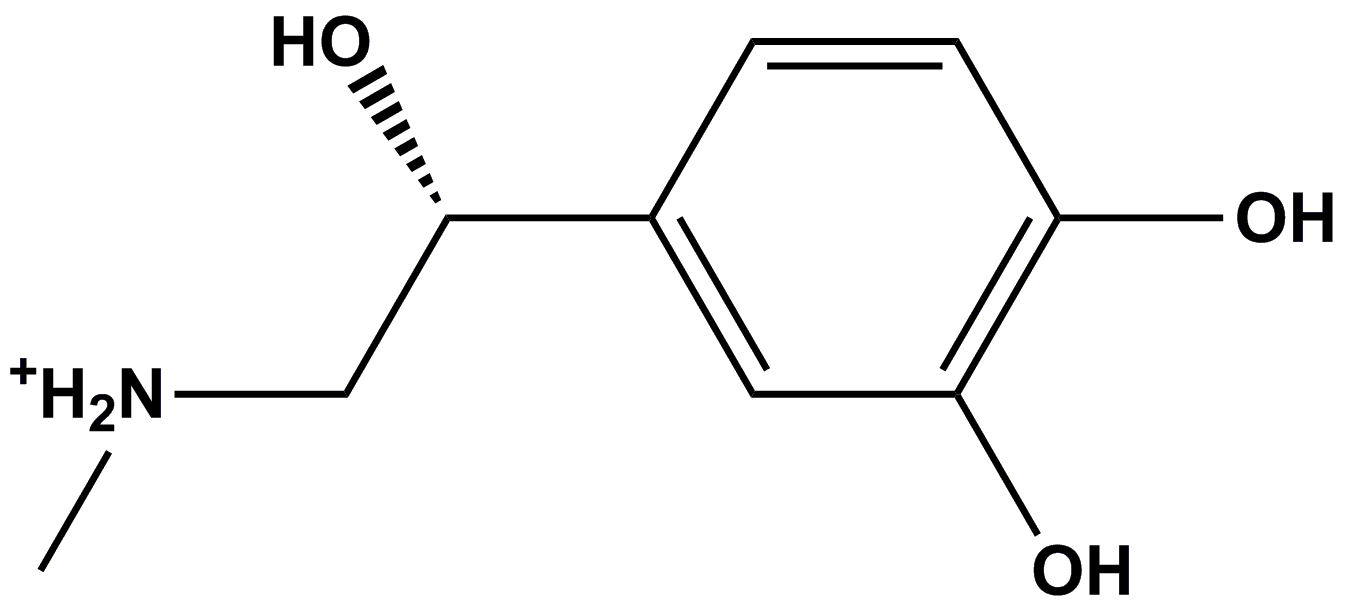 |
| BI-167107 | 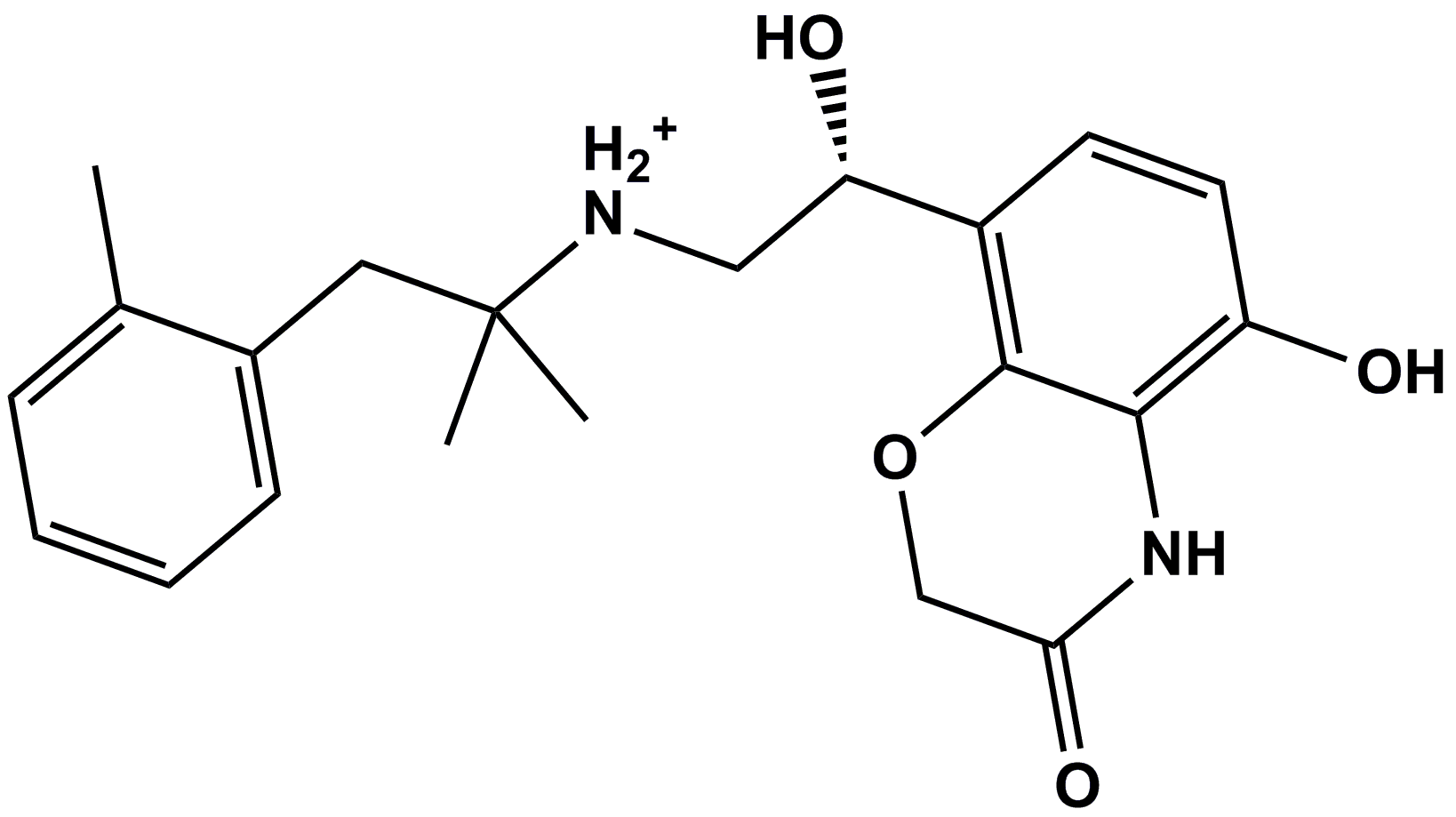 |
| Clenbuterol | 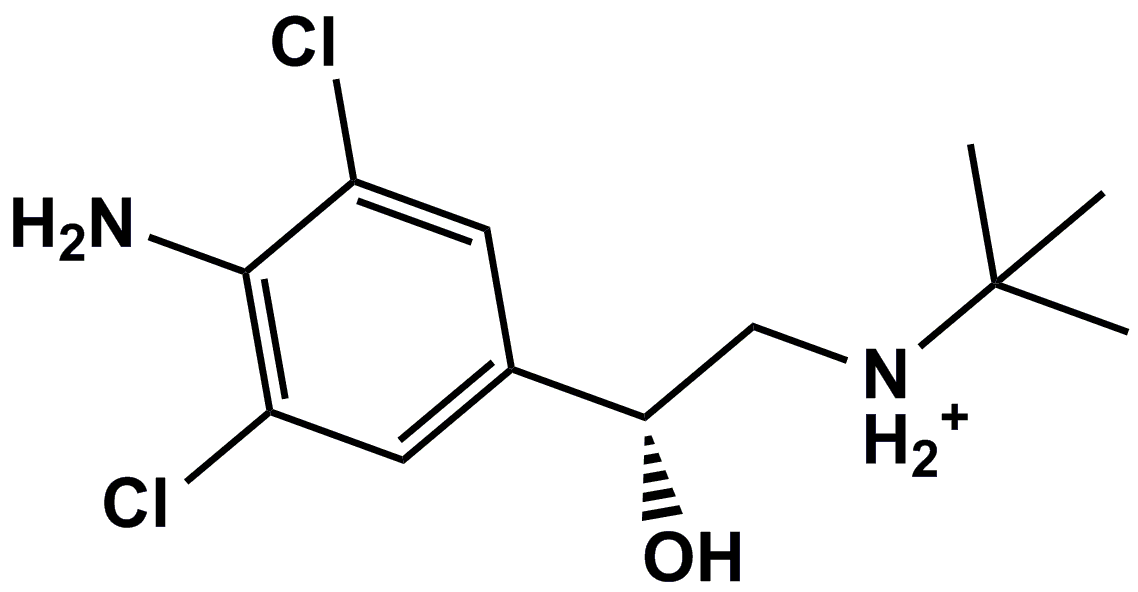 |
| Fenoterol | 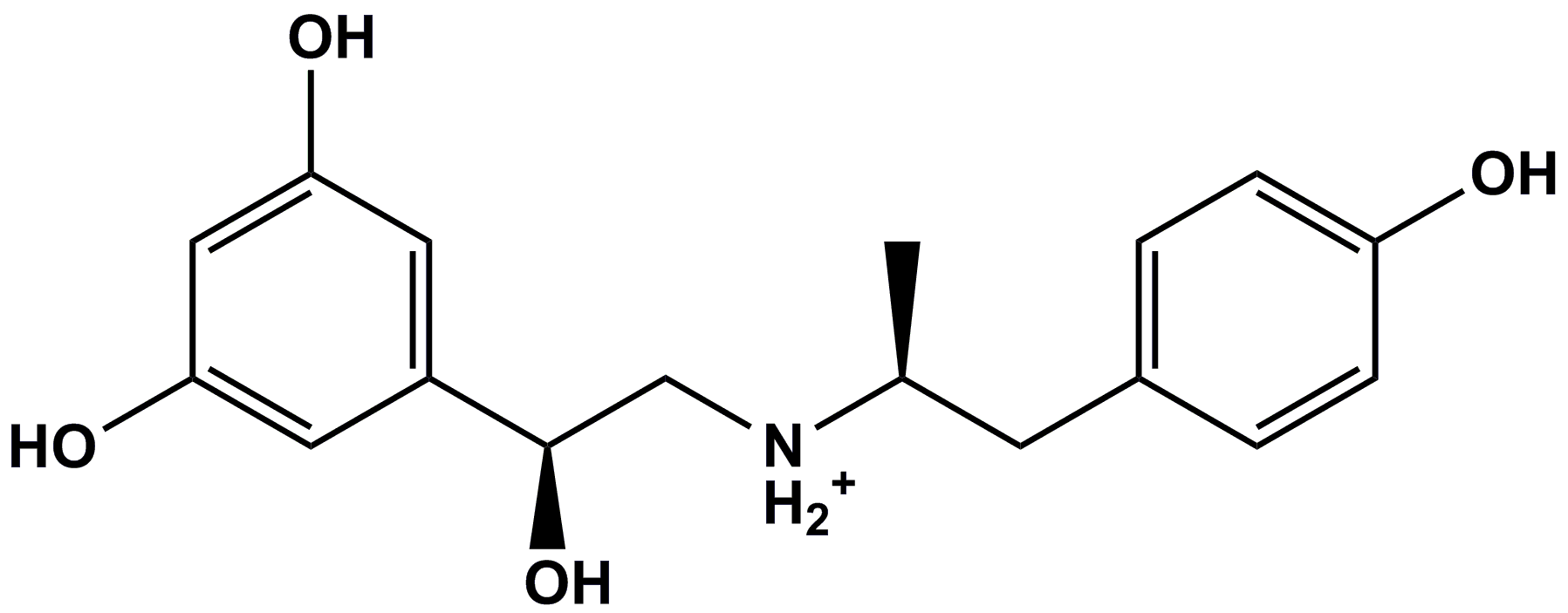 |
| Hydroxybenzyl isoproterenol | 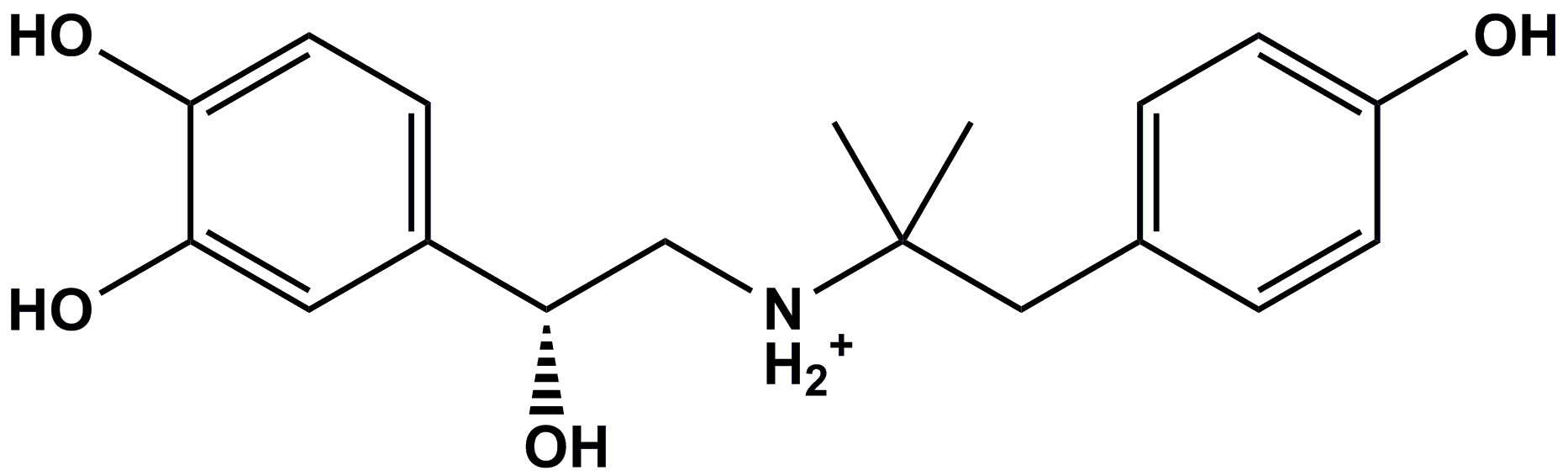 |
| Isoprenaline | 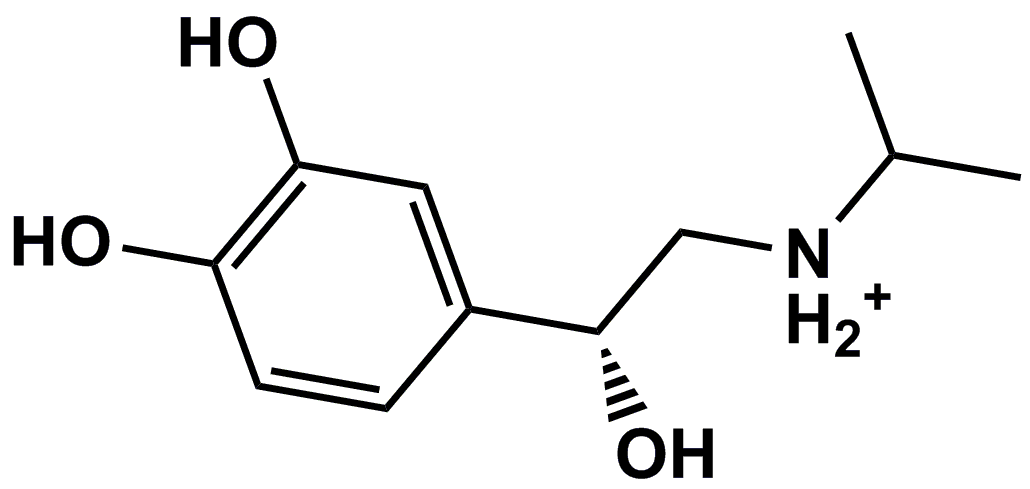 |
| Metaproterenol | 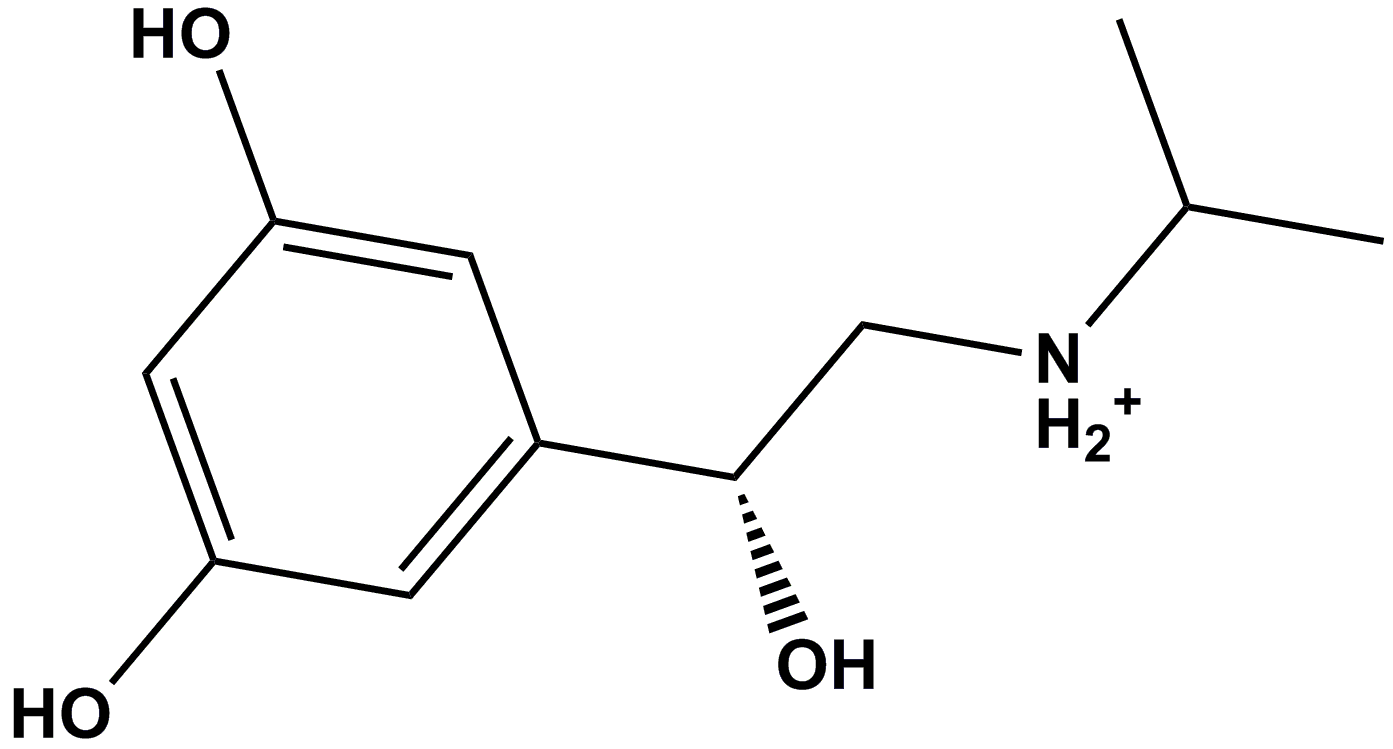 |
| Pirbuterol | 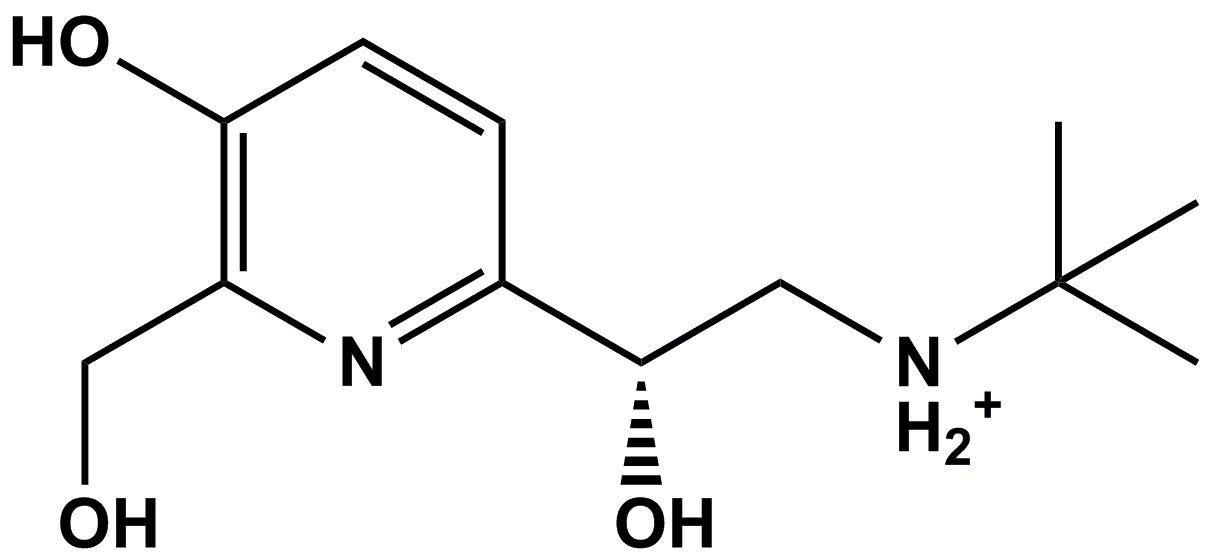 |
| Ritodrine | 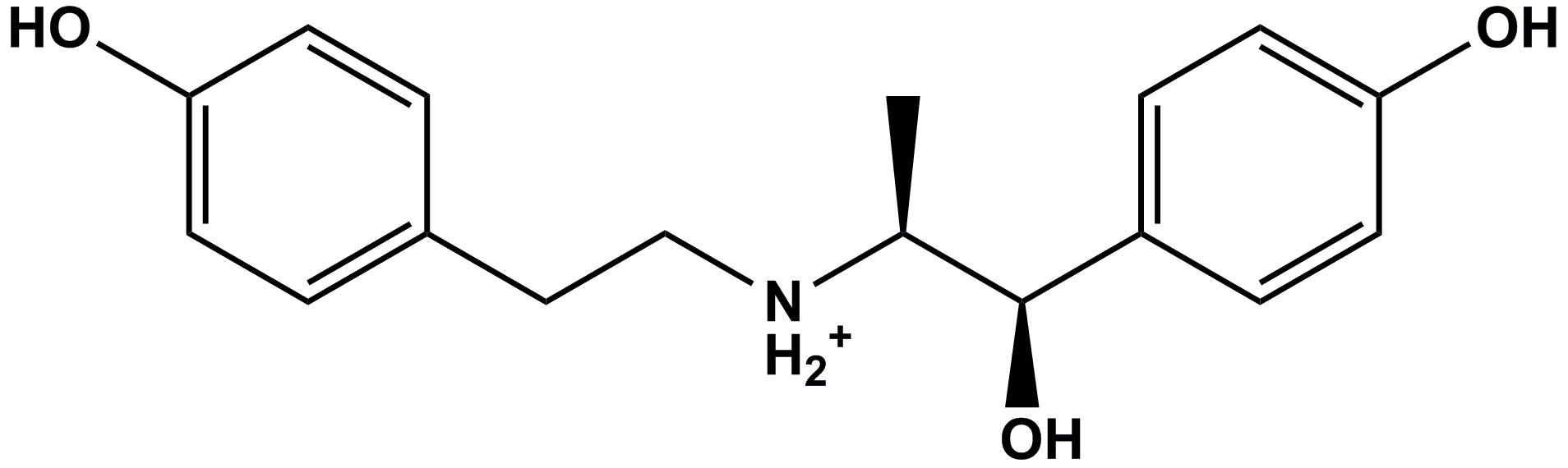 |
| Salbutamol | 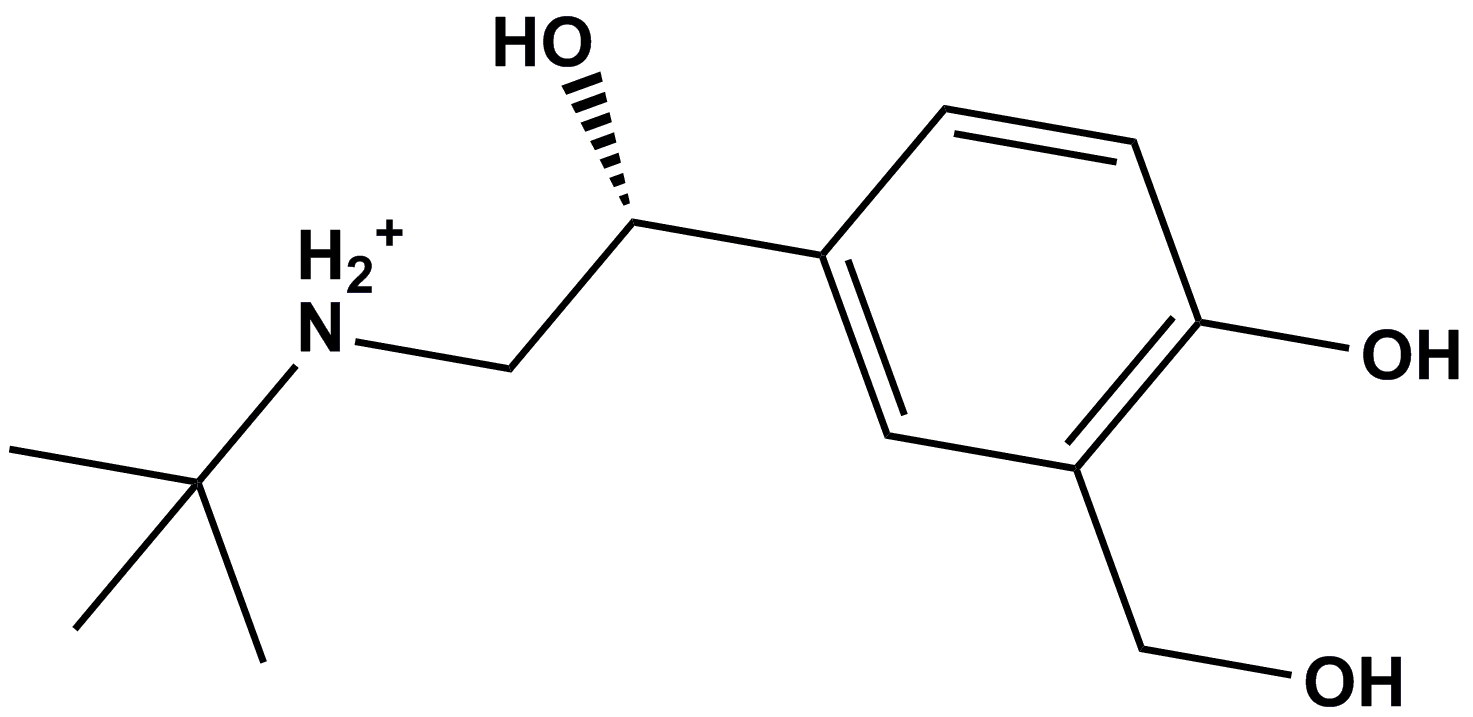 |
| Terbutaline | 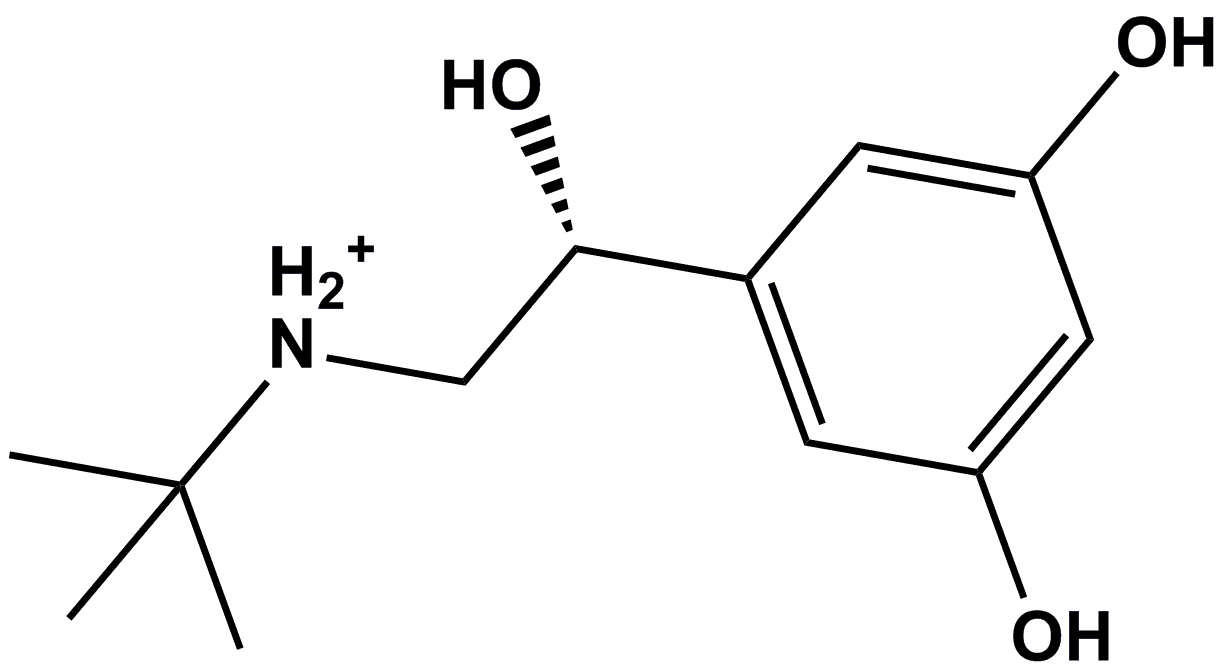 |
